# Editorial: Cardiomyocyte Microdomains: An Emerging Concept of Local Regulation and Remodeling

**DOI:** 10.3389/fphys.2020.00512

**Published:** 2020-05-26

**Authors:** Di Lang, Sarah C. Calaghan, Julia Gorelik, Alexey V. Glukhov

**Affiliations:** ^1^Department of Medicine, University of Wisconsin-Madison School of Medicine and Public Health, Madison, WI, United States; ^2^School of Biomedical Sciences, University of Leeds, Leeds, United Kingdom; ^3^Myocardial Function, National Heart and Lung Institute, Imperial College London, London, United Kingdom

**Keywords:** microdomain, cardiomyocte, caveolae, t-tubule, L-type Ca channel, intercalated disc, Na channel

Technological advances facilitate scientific breakthroughs that lead to leaps in our understanding of human physiology and pathophysiology. We are beginning to recognize the key roles of cardiomyocyte microdomains in essential aspects of many cellular processes and their importance as a mechanistic driver of life-threatening arrhythmias and cardiac dysfunction in a range of pathologies. This concept opens new avenues to understand heart disease from a more systematic and dynamic perspective and benefits clinical treatment strategies, as summarized in the current Research Topic.

Emerging evidence demonstrates that in cardiomyocytes discrete clusters of ion channels, regulatory receptors and various signaling molecules are present throughout the sarcolemma, where they form an interacting network and work together as a part of different macromolecular signaling complexes. It has been recognized that compartmentalized distribution of proteins impacts their function and regulation by various neurohormonal pathways, either via a direct interaction with G-protein coupled receptors or by means of second messengers. In the cardiac myocyte, this enables the specificity, reproducibility, and accuracy of the neurohormonal modulation of numerous cellular processes, including membrane excitability and excitation-contraction coupling (ECC) (reviewed in Radwanski et al.), hormone secretion, gene expression, and the immune response during cellular stress (Chen G. et al.). A series of reviews, perspectives, and original research articles in this Research Topic presents the latest advances on microdomain-specific distribution, functioning, regulation, and remodeling of: ion channels, including L-type Ca^2+^ channels (LTCCs) (Wright et al.), transient receptor potential canonical (TRPC) channels (Wen et al.), potassium inward rectifier channels (Vaidyanathan et al.), sodium channels (Chen X. et al.), regulatory proteins and second messengers (Loucks et al.), mitochondria (Zhou et al.), the inflammasome (Chen G. et al.), fibrosis (Shao et al.). Taken together, these give novel insight into the contribution of microdomains to cellular signaling and cardiac pathology.

The most studied aspect is, probably, a compartmentalized distribution of LTCCs, their functional coupling with sarcoplasmic reticulum Ca^2+^-induced Ca^2+^ release channels, ryanodine receptors (RyRs), and their regulation by β-adrenergic receptors (βARs) (as reviewed in Jones et al.) A recent study by Sanchez-Alonso and colleagues demonstrated that disruption of T-tubule structures in heart failure leads to the redistribution of functional LTCCs from their canonical location in T-tubules to the crest of the sarcolemma, where their open probability is dramatically increased via Ca^2+^-calmodulin kinase II (CaMKII)-mediated phosphorylation. This subsequently elevates the whole-cell L-type Ca^2+^ current (*I*_Ca, L_) window current and results in the development of arrhythmogenic early afterdepolarizations (Sanchez-Alonso et al., 2016). A similar mechanism could be also involved in pathological remodeling in cardiac hypotrophy as reviewed in Tanaka et al.

Importantly, redistributed LTCCs are no longer co-localized with RyRs and thus do not contribute to ECC. These findings indicate that the same protein could behave differently when located in distinct subcellular compartments. This could explain the low therapeutic efficiency and high incidence of side-effects of conventional therapies focused on the targeting of activity of certain proteins. For example, conventional calcium channel blockers are generally felt to be contraindicated in heart failure because of their negatively inotropic effects via inhibiting both peak and window *I*_Ca, L_. Such adverse effects may relate to non-specific targeting of LTCCs. It therefore indicates a need for the development of targeted therapies which take into account a microdomain-specific localization of affected proteins as well as their surrounding molecular partners to achieve the localized effects.

The redistribution of LTCCs in heart failure impairs LTCC-RyR functional coupling leading to both electrical and contractile abnormalities (see review by Jones et al.). It is also accompanied by the disruption of an associated macromolecular signaling complex which includes, besides LTCCs, β_2_ARs, protein kinase A, phosphodiesterase 4, and adenylyl cyclase 5/6, resulting in a blunted response of diseased myocardium to sympathetic stimulation. Using novel Forster Resonance Energy Transfer (FRET)-based biosensors for live cell imaging (the most recent methodological advances of FRET biosensors are reviewed by Dikolayev et al.) as well as sophisticated biochemical, electrophysiological and optical (Jayasinghe et al.) techniques, several groups have shown a disruption of β_2_AR-mediated cyclic adenosine monophosphate (cAMP) signaling in failing ventricular myocytes. From a confined, local increase in healthy myocytes under β_2_AR stimulation, cAMP becomes diffuse throughout the cytosol in failing myocytes (Nikolaev et al., 2010). It subsequently leads to abnormal phosphorylation of contractile proteins and loss of the cardioprotective effect of β_2_ARs which contributes to the heart failure phenotype.

Loss of compartmentalized cAMP signaling has been linked to disruption of caveolae and downregulation of caveolar scaffolding protein caveolin-3 (Calaghan et al., 2008; Wright et al., 2014). A crucial role of caveolin-3 in controlling cAMP signaling microdomains has been recently demonstrated in ventricular myocytes (see a computational study by Loucks et al.), as well as in atrial and sinoatrial node myocytes which lack a developed T-tubule system (reviewed in Lang and Glukhov). In atrial myocytes, caveolin-3 participates in the formation of specialized “axial couplons” that are composed of voluminous axial tubules with extensive junctions to the sarcoplasmic reticulum including clusters of highly phosphorylated RyRs (Brandenburg et al., 2016). This increases the frequency of spontaneous Ca^2+^ releases from RyRs when compared with ventricular myocytes (Glukhov et al., 2015) but facilitates the synchronization of the subcellular Ca^2+^ release and thus contraction in atrial myocytes (Brandenburg et al.). Similar clusters of highly phosphorylated RyRs have been also identified in sinoatrial node pacemaker cells where they are responsible for the generation of spontaneous local Ca^2+^ releases, a critical part of the Ca^2+^ component of the coupled-clock pacemaker system (reviewed in Vinogradova et al.).

Finally, caveolae structures have been described as membrane mechanosensors and linked to the activation of mechano-sensitive, swelling-activated chloride channels *I*_Cl, swell_ (Egorov et al., 2019). Stretch-dependent regulation of membrane microdomains has been proposed to play an important role in the mechano-electrical feedback in the heart regulating pre-loading dependent cardiomyocyte shortening (Kozera et al., 2009), LTCC and β_2_AR signaling microdomains (Wright et al.) and also could be involved in stress-induced membrane damage (Nikouee et al.) and associated inflammation (Chen G. et al.).

## Conclusions and Future Directions

All these studies pave the road to expand our knowledge of cardiac physiology from the new perspective of microdomain compartments which will advance clinical treatment strategies in the future. In most cases, a poor efficacy of current therapeutics can be ascribed to incomplete understanding of electrophysiological, cellular and molecular mechanisms that underlie certain heart diseases. The conventional description of cardiac pathologies, in terms of dysfunction of one or several proteins often described via changes in their expression or function, further compounds the poor efficacy of available therapies. These limitations contribute to the non-specific action of cardiac medications on other organs and tissues, and even throughout different regions of the heart. This leads to common side-effects of conventional therapies and, in the worse cases, increases morbidity and mortality in a long-term perspective.

This view was changed by consideration of post-translational modification of proteins. Various post-translational modifications, including phosphorylation, glycosylation, nitrosylation, and lipidation, have been shown to dramatically modify protein activity, critically contributing to pathological cell signaling. Recent methodological advances have made it possible to visualize localization, regulation and interaction of single proteins at nanoscale resolution allowing study of the clustering of functional ion channels, receptors, and various second messengers as well as their coupling within specific cellular microdomains. This extends the classical concept of cardiac remodeling and adds a new dimension to cardiovascular disease, namely microdomain-targeted protein remodeling, which could be a development platform for more sophisticated therapeutic approaches based on the subcellular distribution of their targets.

## Author Contributions

AG and DL wrote the editorial. SC and JG provided comments and edits.

## Conflict of Interest

The authors declare that the research was conducted in the absence of any commercial or financial relationships that could be construed as a potential conflict of interest.
